# Neurological impairment in long COVID: implications for neurodegenerative disease

**DOI:** 10.1186/s12967-026-08607-y

**Published:** 2026-07-10

**Authors:** Etianne Martini Sasso, Natalie Eaton-Fitch, Kiran Thapaliya, Sonya Marshall-Gradisnik

**Affiliations:** National Centre for Neuroimmunology and Emerging Diseases, Health Group, Griffith University, Gold Coast, Australia

**Keywords:** Long COVID, Post-acute sequelae of SARS-CoV-2, Neurodegenerative diseases, Neurological alterations, COVID-19, Neuroimaging, Neuroplasticity

## Abstract

**Background:**

It has been six years since the COVID-19 pandemic and, despite substantial advances in management, the disease sequelae known as long COVID continues to represent a significant medical and societal burden. Long COVID is characterised by persistent neurological and neurocognitive symptoms, including brain fog, memory deficits, attention impairments, and fatigue, lasting for months after acute SARS-CoV-2 infection.

**Main body:**

In this review, we collated emerging neurological findings related to long COVID, discussing neurodegenerative processes associated with long COVID, potential clinical implications and research limitations. Neurological and neurocognitive manifestations arise through multiple mechanisms, including direct SARS-CoV-2 invasion of the central nervous system and peripheral lymphocyte infiltration. Additionally, neurovascular damage potentially contributes to neurodegeneration through neuronal injury, impaired neurogenesis, microvascular abnormality and sustained neuroinflammation.

**Conclusion:**

Understanding the mechanisms underlying neurological and neurocognitive symptoms is essential for developing long-term monitoring strategies and targeted interventions to mitigate neurocognitive decline in individuals with long COVID.

**Supplementary Information:**

The online version contains supplementary material available at https://doi.org/10.1186/s12967-026-08607-y.

## Introduction

In March 2020, the World Health Organization (WHO) declared a global pandemic caused by severe acute respiratory syndrome coronavirus 2 (SARS-CoV-2), the infectious agent responsible for the highly transmissible respiratory disease called coronavirus disease 2019 (COVID-19) [1, 2]. Given the natural decrease in virulence and transmissibility of the SARS-CoV-2 virus, in conjunction with the rapid response of the scientific community resulting in vaccine development and improvements in clinical care, the impact of acute COVID-19 was successfully reduced in the years following the pandemic [3, 4]. However, there remains a significant and ongoing global challenge due to the impacts caused by long COVID (also known as post COVID-19 condition, post-acute sequelae of COVID-19 and post COVID-19 syndrome) [5–11].

According to epidemiological studies, the prevalence of long COVID is approximately 10% of people infected with SARS-CoV-2 [12, 13]. People with long COVID experience a vast range of multisystemic symptoms that are categorised as neurological, respiratory, cardiovascular, gastrointestinal and immunological in nature [12, 14–19]. Notably, there is a substantial variability in the clinical features and risk factors associated with long COVID, which consequently makes it difficult to define diagnostic criteria and study population [20].

Persistent neurological and neurocognitive alterations are among the most frequently reported symptoms in people with long COVID and contribute significantly to increasing functional impairment and a poor quality of life [12, 21, 22]. The neurological manifestations in people with long COVID raise concerns regarding the risk of neurodegeneration or potentially the heightened risk of developing secondary neurodegenerative diseases. Research has documented potential SARS-CoV-2 neuroinvasion and current knowledge suggests this virus induces neuroinflammation, blood-brain barrier (BBB) disruption, and microvascular damage, thus directly and indirectly affecting the central nervous system (CNS) [11, 23–26]. Notably, the neurocognitive symptoms align with the abovementioned alterations in long COVID emphasising the importance of research focused on investigating the potential association between long COVID and neurodegeneration [27–30].

In this review paper, we explore the current neurological findings of long COVID, integrating evidence of neurodegeneration. Furthermore, we will discuss their clinical applications, along with future research directions. This editorial review incorporates peer-reviewed research identified through targeted search terms according to key research areas in the field, including ‘long COVID AND neurodegeneration’, ‘long COVID degenerative’, ‘post COVID-19 condition AND neurodegeneration’. As long COVID is an emerging disease with only a few years of research available, no restrictions were applied to the publication period. Therefore, studies published between 2020 and 2025 were included in the search. The initial search for manuscripts was conducted in early September 2025 and a final search in the middle of December 2025 to include potential new publications not included in the previous search. PubMed was the main database used and we prioritised original research and articles that included data on long COVID, although we also included a limited number of papers focused on acute COVID-19 to provide contextual and mechanistic insights.

## Neurological impairment in long COVID

The nervous system appears to play a central role in long-term sequelae of COVID-19, as up to 80% of people with long COVID report neurological manifestations [31]. These include fatigue, ‘brain fog’, headaches, loss of senses and sleep disorders that are persistent and potentially severe [22, 31–34]. The term ‘brain fog’ refers to a significant alteration in both long- and short-term memory, concentration, attention, information processing speed and executive function [22, 23, 32, 35]. There are risk factors associated with the lack of complete recovery after acute SARS-CoV-2 infection. These risk factors include the female sex, higher symptom burden during acute SARS-CoV-2 infection, infection with pre-Delta-variants (e.g., Wuhan-Hu-1, D614G, Alpha, Beta, Gamma, Epsilon, Zeta, Eta, and Iota), and absence of vaccination against SARS-CoV-2 before acute infection [36].

Despite risk factors for long COVID being well characterised in the literature, emerging evidence is targeted towards neurological manifestations in this condition. The female sex also has a significantly higher risk for neurological manifestations in long COVID, such as fatigue, neurocognitive deficits, memory impairment, loss of concentration, anosmia, dizziness, headache and psychological symptoms compared to males [37–40]. Although the underlying mechanism for this increase in prevalence in females remains elusive, some hypotheses are related to increased susceptibility of women to an autoimmunity response to COVID-19 spike protein antigen, microbiome difference between genders, potential overexpression of the angiotensin-converting enzyme 2 (ACE2) gene located on X chromosome, and sex hormone differences [41–44]. Age as a risk factor for neurological symptoms in long COVID remains limitedly investigated. Notably, a recent study identified reduced frequency and severity of long COVID in the oldest group (≥ 70 years) in comparison to the youngest adult group (18–39 years), while the increased prevalence was in middle-aged adults’ groups (40–49 years and 50–59 years) [45]. Similarly, another investigation reported that despite older individuals with long COVID group (≥ 65 years) experiencing a high frequency of neurologic manifestations, younger (18–44 years) and middle-aged (45–64 years) patients suffer from a higher burden of neurological symptoms and cognitive dysfunction, regardless of their hospitalisation status [46]. In support of this, cognitive impairment was also associated with a younger patient group in another study [47]. In contrast, these findings diverge from a prior investigation conducted from January 2020 to June 2021, in which researchers identified an association between the older age group and neurological symptoms in long COVID [37]. These divergences are potentially affected by other confounding factors, such as inclusion criteria, SARS-CoV-2 variants and vaccination status.

Although the prevalence of neurocognitive symptoms in long COVID varies across studies, there is consensus regarding the symptoms most commonly reported [48]. A recent investigation by Huff et al. identified the prevalence of fatigue, difficulty with concentration, unrefreshing sleep and dysarthria varying from 73.2% to 86.4% in people with long COVID, while less frequent symptoms, including dizziness/light-headedness, headaches, general weakness, numbness/tingling, myalgia, and focal weakness varied from 51.6% to 61.0% [31]. The authors also reported no significant difference in the prevalence of neurological symptoms between hospitalised or non-hospitalised individuals or those with preexisting neurologic and psychiatric conditions [31]. Weigel et al. reported the high prevalence of neurological symptoms including headaches (80.6%), neurocognitive impairment (concentration, 93.5%; short-term memory loss, 45.2%), sleep-related manifestations (unrefreshed sleep, 93.5%; sleep disturbances, 83.9%), and sensory disturbances (photophobia, 64.5%; sensitivity to noise or vibration, 64.5%) in long COVID [49]. Nehme et al. examined individuals with long COVID and identified fatigue, difficulty in concentration, headache, insomnia, loss or change in smell and/or taste, myalgia, arthralgia, and dyspnoea as the most common ongoing symptoms at 15 months after testing positive for SARS-CoV-2 [21]. A review from Nicotra et al. on neurocognitive sequelae supports the abovementioned findings documenting memory impairment reported in 82% of studies reviewed, attentional complaints in 69% of studies, and their association with anxiety, depression and fatigue (77% − 85%) [48].

Research has consistently shown memory deficits in people with long COVID. A study conducted by Baseler et al. identified a significant reduction in memory scores collected using patient reported outcome measures in the COVID-19 groups stratified by both confirmed and unconfirmed cases of COVID-19 and hospitalised COVID-19 cases, compared to the non-COVID-19 group across all age groups 25 years and over [50]. While memory scores improved after 12 months following initial SARS-CoV-2 infection, they remained lower in people with persistent symptoms associated with long COVID. In a neuroimaging investigation using Stroop task (cognitive task), Baraniuk et al., found long COVID participants showed significantly prolonged response times to perform the cognitive task compared with healthy controls [28]. This finding persisted despite repeat exposure, which suggested impaired cognitive processing and reduced efficiency of the brain’s network communication during problem solving [28]. Furthermore, research has documented that the incidence of cognitive deficits, dementia, epilepsy or seizures was increased two years following acute COVID-19 compared with other respiratory infections [33]. Despite individuals with long COVID reporting mental health concerns, evidence has demonstrated that psychological symptoms are not sufficient to explain the origins of impaired cognitive function [33, 35].

## Potential pathomechanisms of neurodegeneration in long COVID

Neurodegeneration involves neuronal loss, characterised by the progressive death of neuronal cells, accompanied by motor and cognitive impairment, culminating in dysfunction depending on the brain region affected [51]. In the context of long COVID, the biological underpinnings of persistent neurocognitive symptoms and associated neurodegeneration in long COVID are considered multifactorial and under ongoing investigation. At the time of preparing this review, evidence suggests key mechanisms that potentially contribute to neurodegeneration in long COVID include direct viral neuro-invasion, viral latency or reactivation; sustained neuroinflammation and microglial activation; coagulation and microvascular injury; and metabolic dysfunction and abnormal protein aggregation [52–58]. Whilst further research is necessary, the existence of long COVID subgroups exhibiting distinct cognitive or neuroimaging alterations may be explained by the diversity of underlying mechanisms mentioned above. Therefore, understanding these mechanisms is crucial, as each pathway can disrupt neuronal function through distinct yet potentially interacting processes, ultimately contributing to the cognitive deficits and neurological sequelae observed in long COVID patients. In this section, we will discuss potential pathomechanisms by which SARS-CoV-2 infection may contribute to neurodegeneration in long COVID.

### SARS-CoV-2 in the CNS

#### SARS-CoV-2 infiltration

**Fig. 1:**
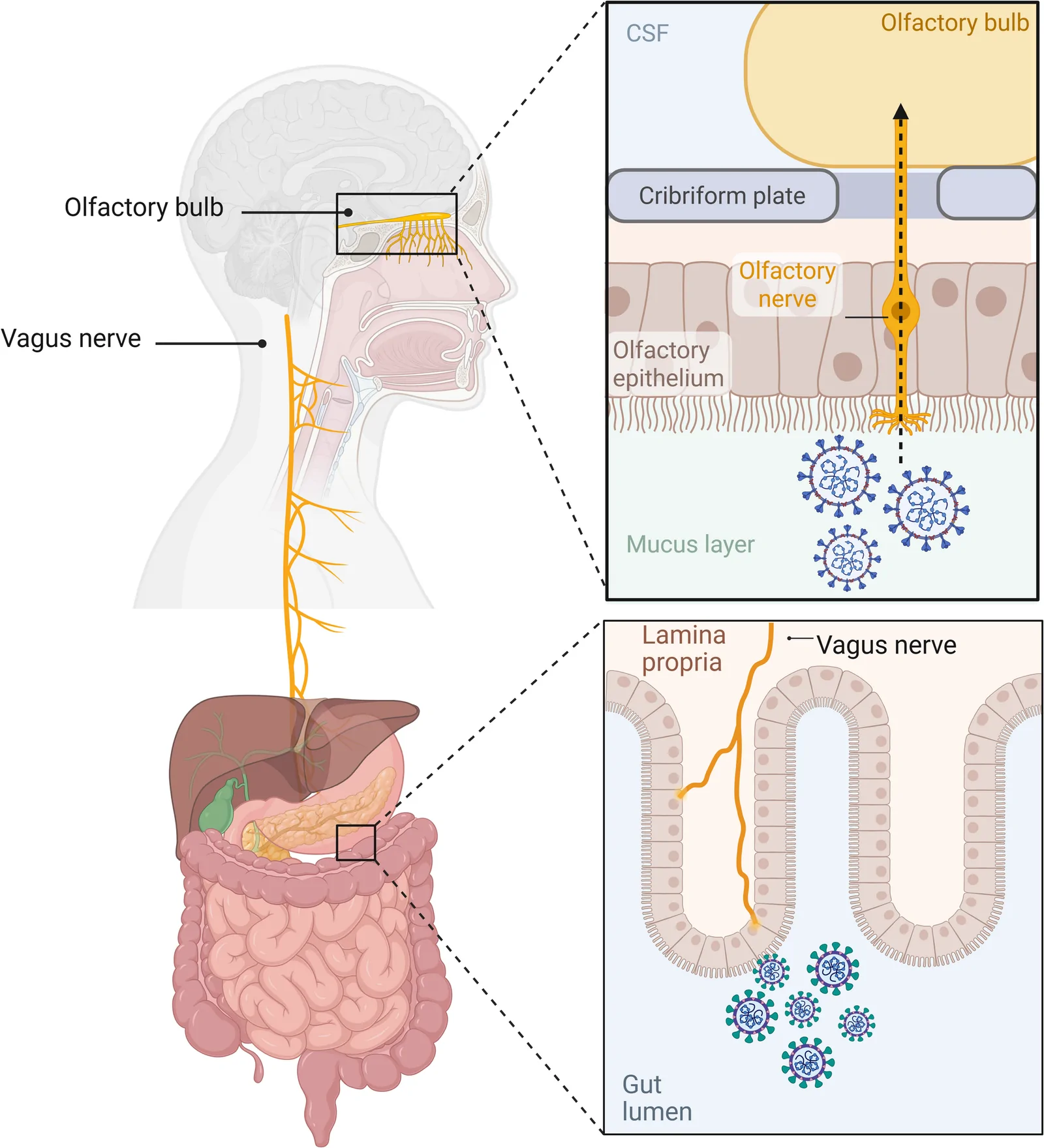
Representation of hypothesised SARS-CoV-2 infiltration of the CNS via the olfactory bulb (top) or the vagus nerve (bottom). SARS-CoV-2 infects cells of the olfactory epithelium layer with the potential to travel along olfactory nerve fibers, crossing the cribriform plate into the olfactory bulb, enabling access to the CNS. Regarding the gut-brain axis route, SARS-CoV-2 infects enterocytes in the intestinal lumen triggering local inflammation and viral persistence. It is hypothesised that the virus travels along the vagus nerve which connects the gut to the brainstem nuclei, providing a pathway for CNS infiltration. Illustration created by the authors using BioRender adapted from [11, 93, 94]

Although infections often occur in peripheral tissues, infiltration into the peripheral and the CNS is postulated (Fig. 1) [32, 57]. Numerous pathways of neuroinvasion are outlined in research; however, the basic principles include the direct infection of nerves in contact with peripheral tissues, infected cells arising from the circulatory system, and rupture or damage of the BBB [57]. Following infection of the olfactory mucosa, SARS-CoV-2 may spread by exploiting the proximity of epithelial, vascular, and neural structures, potentially creating a way for nerve invasion [59].

Coronaviruses manipulate the cell machinery through their interactions with the host cell to complete the viral replication cycle and drive viral pathogenesis. Viruses interfere with signalling pathways that regulate vital cellular processes including DNA repair and replication, immune response regulation, transcription, metabolism and cell cycle modulation [60]. By disrupting these pathways, SARS-CoV-2 may induce cellular disturbances contributing to chronic inflammation and impaired neuronal activity that are characteristics of neurodegeneration [60]. SARS-CoV-2 is a spherical virion of a diameter of 80 to 120 nm formed by a lipid plasma membrane and is recovered by transmembrane proteins that facilitate the attachment of the virus to the host cell [25, 61]. The Spike (S) protein present in the virus plasma membrane interacts with and cleaves host cell receptors, ACE2 and transmembrane serine protease 2 (TMPRSS2) [25, 61]. The presence of ACE2 in the membrane of systemic vascular endothelial cells provides access of SARS-CoV-2 via the BBB. This process is further facilitated by neuroinflammation which increases the permeability of the BBB [25, 62]. These S proteins undergo structural changes influencing permeability of host cell membranes and, consequently, the transmission rate of SARS-CoV-2 variants. The ACE2 receptor is expressed in cells of multiple organ systems, characterising it as the key entry receptor for SARS-CoV-2, while TMPRSS2 is the host factor that facilitates viral uptake and membrane fusion [22]. The SARS-CoV-2 replication cycle initiates with the binding of the viral S protein and the ACE2 transmembrane receptor. This ligation is followed by activation of SARS-CoV-2 S proteins through cleavage by TMPRSS2, facilitating viral entry and triggering innate and adaptive immune responses via cytokine release [63–66].

Researchers have identified the ACE2 receptor and the spike protein protease TMPRSS2 in human olfactory epithelium sustentacular cells, horizontal basal cells that act as stem cells, and Bowman’s gland cells [67]. In addition, ACE2 receptor and TMPRSS2 were detected in the corneal epithelium, and SARS-CoV-2 has been found in various ocular regions, suggesting potential mechanisms of neuronal entry [68, 69]. In a SARS-CoV-2 infection in mice model, damage was identified in the BBB and blood-cerebrospinal fluid barrier at the choroid plexus after SARS-CoV-2 infection, indicating the virus causes pericyte injury, tight-junction disruption, endothelial activation, and vascular inflammation, which leads to microvascular and barrier impairment [70].

While numerous peripheral neurons show the presence of SARS-CoV-2 ribonucleic acid (RNA) [71], another emerging pathway for SARS-CoV-2 entry into the CNS is through the gut-brain axis. SARS-CoV-2 disrupts the gut-microbiome leading to dysbiosis and increased gastrointestinal permeability, resulting in systemic inflammation [72, 73]. While the bi-directional communication between the gut and brain has been investigated in relation to neuroinflammation and clinical manifestations due to altered cytokine regulation and neurotransmitter production, the role of this axis in viral infiltration involves enteric nerves, such as the vagus nerve [74–76]. This nerve is located in different layers of the gastrointestinal wall, which is constantly stimulated by metabolites and bacteria to maintain balance between the brain and the intestinal tract [76, 77]. Therefore, the vagus nerve provides both a functional and physical connection between the gastrointestinal tract and the CNS. It’s postulated that SARS-CoV-2 can infect enteric neurons or intestinal epithelial cells expressing ACE2 receptor and TMPRSS2. Research by Woo et al. documented findings of infiltrating monocytes and CD8 + T cells around the vagal brainstem nuclei of deceased COVID-19 [78]. Additionally, Polymerase Chain Reaction (PCR) of cervical vagal nerve tissue found SARS-CoV-2 RNA in specimens from deceased COVID-19 individuals. Subsequently, viral particles and inflammatory signals may travel along the vagus nerve by passing the BBB for the CNS entry [79, 80].

Supporting evidence from independent studies has demonstrated the presence of SARS-CoV-2 in multiple anatomic sites, including the CNS up to 230 days after the onset of acute infection [26]. Post-mortem examinations indicated presence of SARS-CoV-2 is not limited to respiratory system tissues, but also identified in kidneys, heart, liver, pancreas, endothelial cells, blood and brain [26, 81–83]. There are numerous bodies of research identifying potential pathways by which SARS-CoV-2 may infiltrate the CNS, raising concerns about subsequent neurodegenerative processes. Given the expression of ACE2 receptor in the CNS, neuroinfiltration remains a significant consideration for long-term sequelae, COVID-19 severity, and clinical management. SARS-CoV-2 infection of CNS tissues may amplify neuroinflammatory and immune responses during COVID-19 contributing to neuronal cell loss associated with neurocognitive symptoms reported in long COVID [84].

Although some studies report neuro-invasion through the detection of SARS-CoV-2 in the CNS [26, 81, 85–87], other investigations did not detect viral presence [88–90], which may be attributed to technical challenges in processing post-mortem tissue, analysis of different CNS regions, COVID-19 duration and severity, variant of viruses, viral material may be present at levels below the assay limit of detection or the absence of replication-competent virus in most CNS specimens. Therefore, interpretation of SARS-CoV-2 detection in the CNS and cerebrospinal fluid of COVID-19 and long COVID patients requires caution. Direct CNS invasion by SARS-CoV-2 continues to be a matter of considerable debate, as the presence of viral material does not provide definitive evidence of active infection or establish a causal association with neurodegeneration [88, 91, 92]. 

Overall, the consequences of SARS-CoV-2 infiltration in the CNS through multiple pathways, including the olfactory nerve, vagus nerve (gut-brain axis), and ventricular pathways may have significant implications for the development and potentially the severity of long COVID. Persistent SARS-CoV-2 viral particles in neural tissues may induce chronic neuroinflammation, disrupt the BBB and impede neuronal signalling. These processes are subsequently linked with microglial activation, neurovascular injury and neurocognitive impairment reported in long COVID [95–98]. Therefore, it is hypothesised that the abovementioned neuroinfiltration mechanisms could potentially lead to acute infection, viral infiltration and cause long-term sequelae of neurocognitive disturbances in long COVID.

#### Viral latency or reactivation

Another widely investigated pathomechanism potentially implicated in neurodegeneration among people with long COVID includes reactivation of infections, such as Epstein-Barr virus (EBV), cytomegalovirus (CMV), herpes simplex virus (HSV), and varicella zoster virus [20, 99, 100]. Recent EBV reactivation and preexisting HIV infection were independently associated with increased prevalence of long COVID symptoms [52].

Proposed mechanisms of long COVID with viral reactivation include chronic inflammatory responses, alterations in cytokine production, dysregulation of immune responses and tissue damage caused by reinfection [12, 65, 101, 102]. As an example, EBV reactivation has been proposed in COVID-19 patients [99]. The role of EBV infection in neurological disorders has been previously discussed with emphasis on the development of multiple sclerosis [103, 104] and Myalgic Encephalomyelitis/Chronic Fatigue Syndrome (ME/CFS) [105, 106] where both conditions share overlapping symptoms with long COVID [7, 107–111]. Studies in EBV have also shown disruption to BBB permeability and neuroinflammation [104].

Despite activation of latent infection being a factor potentially associated to increase the risk of developing long COVID, it is not an essential factor for all individuals to develop long COVID [52]. In fact, previously detected CMV infection was linked to reduced occurrence of neurocognitive dysfunction and also to a lower prevalence of long COVID [52]. Although this finding is preliminary and requires further investigation, it has been hypothesised that the CMV protective pathway is based on a robust immune response to SARS-CoV-2 or the occurrence of virus-induced immunoregulatory pathways, such as IL-10 activation and signalling [112]. These conflicting findings regarding the role of viral persistence in long COVID emphasise the complexity of viral-host interactions and highlight the need for continued investigations to address gaps in the understanding of this emerging condition.

Regarding SARS-CoV-2, there is evidence to suggest that the virus may persist in tissue reservoirs, including brain, liver, intestine, stomach, kidneys, intestine, lung, breast, skin, thyroid, adipose and vascular tissue, months after the acute infection [113–116]. Specifically, an investigation conducted by Swank et al. detected SARS-CoV-2 S proteins in approximately 60% of long COVID participants from two to 12 months after acute infection, in contrast, the S protein was not detected in the cohort of COVID-19 recovered participants [117]. Persistent tissue reservoirs of SARS-CoV-2 may contribute to long-term sequelae and predispose a person with long COVID and neurodegeneration. The occurrence of neurocognitive symptoms in long COVID is associated with sustained immune dysregulation and vascular dysfunction due to persistent spike superantigen-like motifs that can skew T-cell receptor repertoires [118]. This may result in T cell and natural killer (NK) cell exhaustion in long COVID, as well as immune cell infiltration of the CNS and microglial activation [85, 95–98]. S protein persistence has been linked to disruption of pericytes, endothelial cells, and BBB integrity and consequently results in neurovascular dysfunction, neuronal damage and neurocognitive deficits [56, 119, 120]. This is supported by experiments in mice revealing that S proteins induced synapse damage and memory impairment [121]. These effects may promote chronic neurovascular dysfunction, cellular exhaustion, or a refractory inflammatory state, providing a likely contributing mechanism for ongoing cognitive impairment in long COVID.

Viral latency is an opportunity for prolonged immune response and chronic inflammation, disruption to innate and adaptive immune responses and risk of neurodegeneration similar to multiple sclerosis. Further research may aim to understand the link between viral reactivation and its effect on the neurocognitive symptoms experience by long COVID.

### Neuroinflammation

**Fig. 2:**
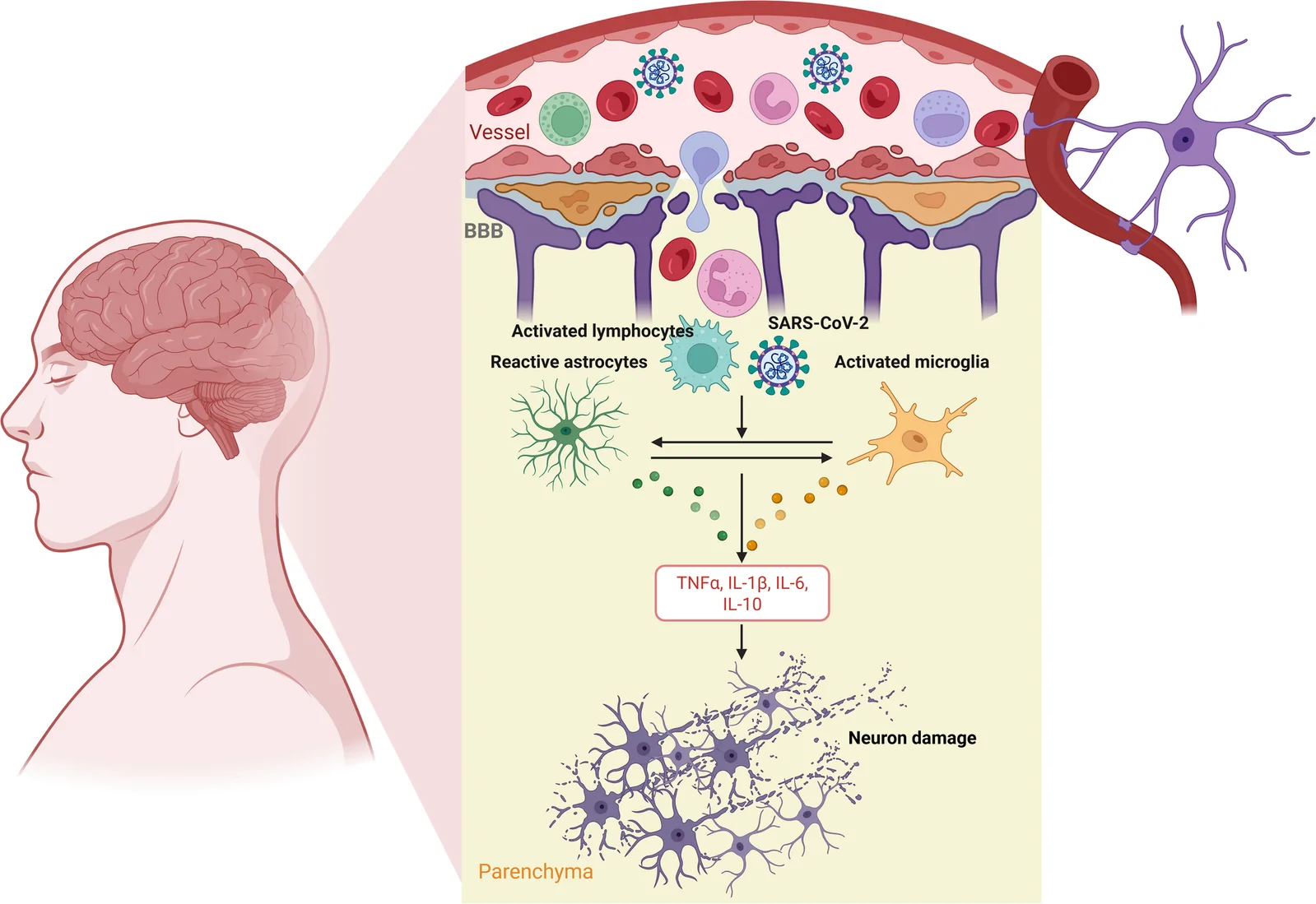
Proposed mechanisms of neuroinflammation in SARS-CoV-2 infection and associated cognitive disturbances and potential neurodegeneration in long COVID. Persistence of SARS-CoV-2 neuroinfiltration and increased BBB permeability might result in activation of glial cells and infiltration of peripheral leukocytes and lymphocytes contributing to increased pro-inflammatory cytokine release contributing to loss of myelinated axons and neuronal damage. The top of the illustration demonstrates circulating lymphocytes and leukocytes in a meningeal capillary before infiltration through the BBB. Illustrated by the authors using BioRender adapted from [128, 129].

In the context of long COVID, neuroinflammation is frequently suggested as one of the key contributors to neurodegenerative processes. Figure 2 depicts potential mechanisms involved in neuroinflammation and neurodegeneration contributing to long COVID. In chronic conditions, neuroinflammation is potentially sustained, contributing to a perpetuating cycle of neuronal damage and degeneration [122]. Chronic neuroinflammation is characterised by excessive activation of microglia and astrocytes which release pro-inflammatory cytokines and chemokines [123]. Neuroinflammation in long COVID is potentially initiated by activation of glial and endothelial cells and plays a protective role during early and brief inflammatory responses. It is hypothesised that prolonged neuroinflammation may lead to neurological damage that has been linked to conditions such as Alzheimer’s disease, Parkinson’s disease, ME/CFS and multiple sclerosis; however, further investigations are necessary to confirm these associations [124–127]. Evidence of neuroinflammation has been reported in long COVID and the underlying mechanism will be elaborated in the subsequent sections.

#### Microglial and astrocyte activation

Microglial cells and astrocytes are subtypes of glial cells situated in the brain with both anti- and pro-inflammatory mechanisms. The chronic activation of these glial cells consequently results in neuroinflammation through dysregulation of neuronal synaptic activity and impaired myelin homeostasis, disturbs neuroplasticity and hippocampal neurogenesis, and impedes cognitive processes [11, 23, 130]. Microglia cells play a crucial role in immune defence and the maintenance of brain homeostasis through functions such as injury repair, intercellular communication and the clearing of apoptotic and necrotic cellular debris. However, in response to neuronal injury, microglia become activated and release cytokines and chemokines [124, 131–133]. An investigation by Olajide et al. reported that SARS-CoV-2 proteins activate nuclear factor kappa B that induced nucleotide-binding domain like protein 3 (NLRP3) inflammasome-mediated pro-inflammatory responses in a mouse microglial cell line [53]. Furthermore, post-mortem examination of brain specimens of people with COVID-19 showed significant microglial activation [85, 89, 134–136]. For this reason, the dysregulated microglial activation causes excessive neuroinflammation and microglia activation has been associated with neurodegenerative diseases [124, 131–133, 137].

Early research hypothesised that SARS-CoV-2 neuroinfiltration, namely mediated through astrocytes, may lead to proinflammatory responses triggering inflammatory dementia-like related pathological changes [138]. Astrocytes are key responders to neuroinflammatory changes via pro- and anti-inflammatory actions. Horowitz et al. hypothesise that hypometabolism observed in people with long COVID using positron emission tomography (PET) is a signature of astrocyte-regulated glutamatergic dysregulation [139]. This finding is also supported by Segabinazi et al. [140]. In brain organoid models, astrocytes showed the highest rate of SARS-CoV-2 infection in brain tissue [138]. Astrocytes derived from human induced pluripotent stem cells (hiPSC) demonstrated susceptibility to SARS-CoV-2 infection, triggering apoptosis of infected astrocytes, eliciting pro-inflammatory responses shown by increased interleukins (IL) including IL-6, IL-4 and IL-15 [140]. Thapaliya et al. recently reported on the elevation of glutamate levels in the posterior cingulate cortex of the long COVID cohort [141]. These findings are aligned with increased inflammatory cytokines and activated microglia, which was associated to an increase in glutamate release, transmission and metabolism, and consequently reduces the number of glutamate receptors on astrocytes and activity of glutamate synthetase [142]. This positive feedback elevated glutamate and may contribute to multiple symptoms experienced by people with long COVID. Interestingly, reduced glutamate causes depleted energy, increased levels induce cell death, and normal levels are essential for memory, cognition, neuroplasticity and mood regulation [143, 144].

#### Peripheral immune infiltration

The activation of microglia and astrocytes consequently disrupts the BBB and is associated with increased permeability and subsequent infiltration of peripheral immune cells into the CNS, a phenomenon commonly reported in multiple sclerosis and Alzheimer’s disease [145–148]. Activated glial cells release chemokines and cytokines, attracting peripheral immune cells, where their infiltration in the CNS exacerbates neuroinflammation [122, 149, 150]. An investigation by Etter et al. reported on peripheral immune mediators in cerebrospinal fluid (CSF) demonstrating increased BBB permeability, reactive B cells, and microglial activation associated with COVID-19 neurological sequelae [54]. Research suggests that CSF levels of pro-inflammatory proteins and the absence of anti-inflammatory mediators are potential predictors of long COVID [54, 151]. Further, postmortem examination of deceased COVID-19 patients has observed brain-wide infiltration of CD8 + T cells [152]. While an investigation in participants two years post acute SARS-CoV-2 infection using PET found activated T cells in the brainstem and spinal cord [153]. Taken together, these findings indicate persistent neuroinflammation in the CNS, which can increase pro-inflammatory cytokines and sustained immune cell activation. Chronic immune activation may impair neuronal homeostasis, promote excitotoxicity and trigger microglial-mediated damage, ultimately contributing to progressive neurodegenerative processes observed in long COVID [102, 154, 155]. Understanding the pathomechanisms and interactions between the CNS and peripheral immune systems in neurodegenerative diseases, as well as in long COVID, provides potential therapeutic pathways to improve health outcomes.

#### Cytokine storm

The strong immune response during acute SARS-CoV-2 infection in the respiratory system leads to a neuroinflammatory condition that, in conjunction with cytokine storm, also increases the chemokines and immune cell trafficking in the CNS [11, 23]. The phenomenon known as cytokine storm is one of the mechanisms associated with the intense immune response observed during acute COVID-19 [23, 156, 157]. It consists of a significant increase in cytokines, which has been associated with severe cases of COVID-19 [65, 102, 158]. Cytokine storm is a key factor responsible for SARS-CoV-2-induced respiratory tissue damage, respiratory distress and patient death [11, 156]. Genetic analysis detected that people with long COVID have dysregulated expression of genes involved in cytokine signalling, immune cell activation and antigen presentation, which might also contribute to the cytokine storm [102].

Emerging studies reported that the dysregulation in cytokine levels is not restricted to the acute COVID-19 period. For example, Schultheiβ et al. reported a correlation between long COVID and a noticeable increase in plasma levels of IL-1β, IL-6, and tumour necrosis factor (TNF), likely secreted by overactivated monocytes and macrophages [159]. Similarly, another study showed that long COVID patients exhibited elevated levels of cytokines, including TNF-α, interferon-γ–induced protein 10 (IP-10), and IL-6 levels suggesting they may represent important risk factors for long COVID [160]. Regarding neurodegeneration, elevated peripheral cytokine levels can permeate the BBB, initiating the neuroinflammatory response directly or through compromising BBB integrity, which opens access to infected leukocytes into the CNS further perpetuating excessive cytokine release, microglial activation, neuroinflammation and imbalance in neurochemicals [141, 142, 161, 162]. The neuro-invasion of SARS-CoV-2, combined with peripheral leukocyte activation, may lead to elevated pro-inflammatory cytokines, which subsequently trigger a cascade of deleterious events in the brain, triggering neuroinflammation and may be linked to promote neurodegeneration [156].

### Coagulation and vascular damage

**Fig. 3:**
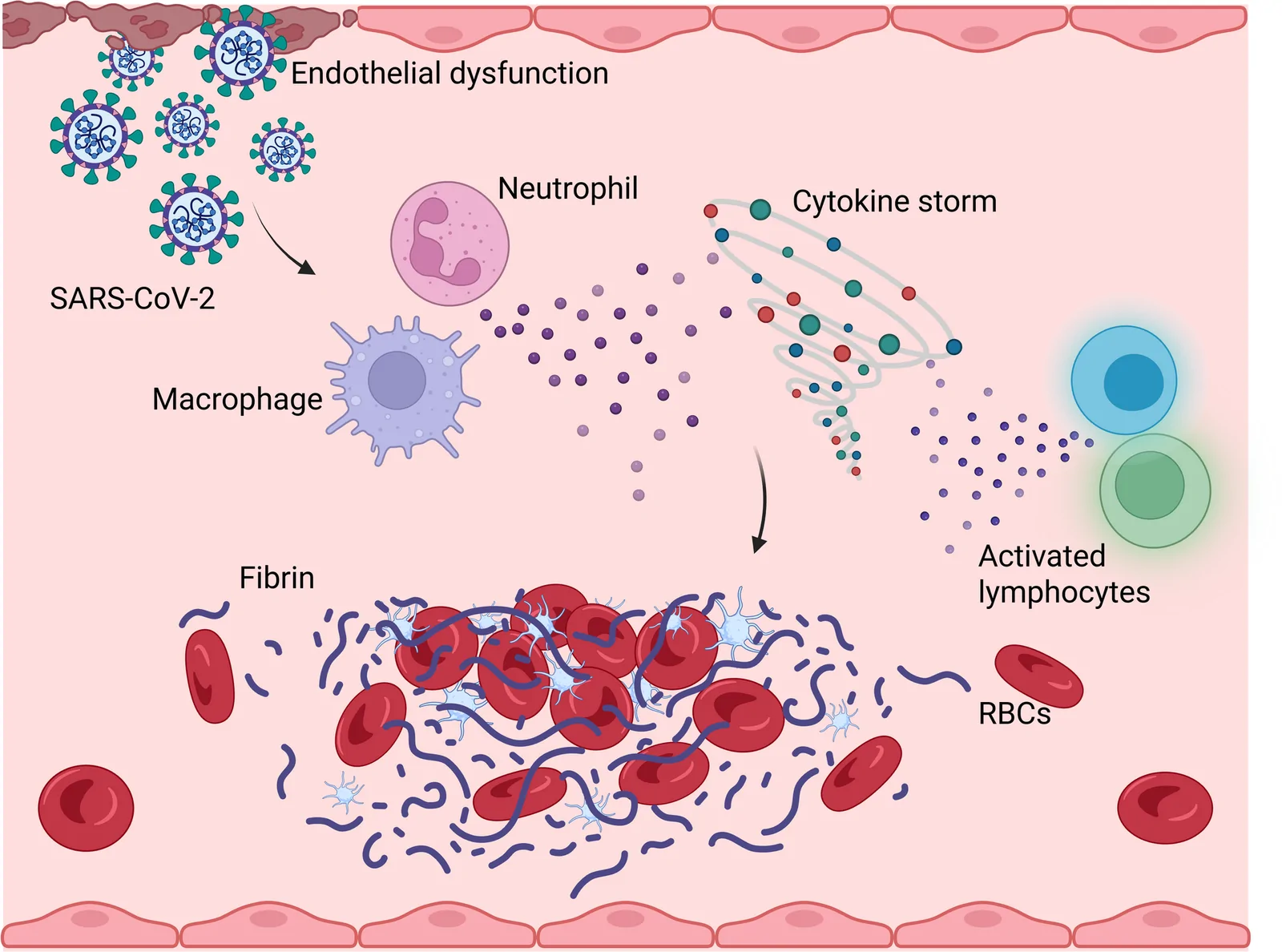
Endothelial dysfunction and immune-mediated coagulopathy during COVID-19 begins with SARS-CoV-2 ligation with ACE2 receptors triggering endothelial activation and disruption. Immune activation results drive cytokine storm through secretion of inflammatory cytokines including IL-1β and TGF-β. Hyperinflammatory state promotes fibrin deposition amplifying immune-mediated coagulation. Persistent endothelial dysfunction contributes to impaired BBB integrity (Fig. 2) contributing to neuroinflammation and neurodegeneration in long COVID. Illustration by authors using BioRender adapted from [176]

Cerebrovascular complications have been reported during acute COVID-19. Indeed, research has demonstrated SARS-CoV-2 can induce apoptosis of brain endothelial cells [163]. A phenomenon also reported during other viral infections including dengue fever virus, influenza virus and HIV [164]. Endothelial damage is hypothesised to contribute to long COVID neurological manifestations due to the potential reduction in blood flow, BBB’s damage, hypoxemia, hypotension, neuroinflammation, and microscopic vascular lesions that directly damage neural networks leading to impaired cognitive processes [23, 24, 165]. Hence, COVID-19 patients are at risk of cerebral vasculitis, thrombosis, microhemorrhages, and ischemic lesions in subcortical and deep white matter, which reduce cerebral perfusion, disrupt the BBB, and allow neurotoxic substances to enter the brain, further aggravating neuronal injury [165, 166]. The cascade of events from SARS-CoV-2 infection to endothelial apoptosis and BBB disruption are associated with neuroinflammation processes and demonstrates a potential pathway of neurodegeneration in long COVID (Fig. 3).

BBB is formed by endothelial cells and supported by astrocytes, pericytes, microglia, neurons, and the basement membrane, relies on tight junctions and specialised transporters to maintain separation between blood and brain and regulate molecular trafficking [55, 56]. As mentioned earlier, the BBB disruption and increased permeability may lead to pathogenic agent penetration and consequently neurological symptoms [25, 57, 62]. Additionally, endothelial damage in long COVID is associated with small fibrin amyloid deposits in the bloodstream of people with long COVID and is also correlated with cognitive deficits [98, 167, 168]. In conjunction with complement activation and complement-mediated coagulopathy, endothelial cell injury are potential pathways involved in the formation of microbleeds or clots [169–171]. Hypoxia and microvascular damage may result in the vascular endothelial injury reported in vulnerable brain regions, including cortex, hippocampus, thalamus, cerebellum, and watershed territories [172–174]. Post-mortem brain samples from people with COVID-19 show increased transforming growth factor-β (TGFβ) signalling and abnormal modification of proteins normally associated with Alzheimer’s disease, compared with non-infected controls [175]. Greene et al. used dynamic contrast-enhanced (DCE) magnetic resonance imaging (MRI) to measure BBB permeability combined with transcriptomic analysis of peripheral blood mononuclear cells (PBMCs) of people with long COVID [24]. DCE-MRI approach suggested BBB disruption in people with long COVID [24]. PBMCs from people with long COVID revealed increased adhesion to human brain endothelial cells in vitro, and exposure of endothelial cells to long COVID serum may induce elevated inflammatory marker expression [24]. Significantly increased inflammatory markers, including IL-1RA, IL-1β, bFGF, IL-13, TGFβ, Glial Fibrillary Acidic Protein, and coagulation markers such as proteins C and S were also reported [24]. Overall, these findings indicate that persistent systemic inflammation and localised BBB dysfunction might be important components of neuroinflammation in long COVID potentially leading to neurocognitive impairment.

## Neuroimaging findings

Research employing objective techniques including PET and MRI have documented potential neurodegeneration, brain injury and functional alterations in individuals with long COVID [139, 177–179]. Data obtained from PET has indicated insights into brain changes in long COVID patients, including reduced brain activity associated with neurological symptoms such as brain fog [139]. In early 2021, Guedj et al. proposed a profile of brain PET hypometabolism in long COVID patients reporting persistent functional impairment; their findings were specific to fronto-orbital and olfactory regions, other limbic/paralimbic regions, the brainstem, and the cerebellum [180]. Subsequently, a multicentre study assessed PET scans from 143 individuals with long COVID with neurological symptoms and reported 53% brain scans as normal, 21% as mildly to moderately or incompletely affected, and 26% as severely affected in line with the COVID hypometabolic pattern [181]. More recent data from Martini et al. observed hyperconnectivity in executive, anterior salience, limbic and attentive networks during an acute/subacute phase [182]. However, in the long term (7 to 12 months) only hyperconnectivity was observed between the executive and attentive networks, aligning with the symptoms of long COVID patients [182]. The hypometabolism observed in people with long COVID is likely associated with astrocyte-related glutamatergic dysregulation and evidence points to therapeutic intervention targeting astrocytic glutamate regulation which may improve long COVID neurological symptoms [139]. These findings were previously discussed in relation to the pathomechanism of neuroinflammation in long COVID.

Using the MRI technique, diverse investigations have detected several neuroimaging alterations in patients after COVID-19. For example, in a longitudinal study, SARS-CoV-2 cases were compared with controls showing greater reductions observed in grey matter thickness and tissue contrast in the orbitofrontal, along with substantial alterations in tissue damage markers in regions functionally linked to the primary olfactory cortex, as well as a greater reduction in the overall brain size [183]. According to an investigation by Samanci et al., long COVID associated cognitive impairments were quantifiable using a series of objective and patient-reported outcome measures. A reduction in grey matter concentrations in the left inferior temporal gyrus, left fusiform gyrus, and right orbital gyri was also observed in the COVID-19 groups relative to controls, as assessed by MRI [184]. The findings demonstrated delayed recall, attention, memory, and visuospatial skills in people with COVID-19 sequelae reporting brain fog when compared to both convalescent COVID-19 patients with or without brain fog and controls [184]. Reduction in grey matter volumes was further confirmed in other studies with COVID-19 recovered individuals [185, 186]. Notably, the results of the study were observed independent of COVID-19 severity, highlighting that cognitive and neurological consequences of long COVID occur even after mild acute infection.

Subsequently, studies focused on long COVID cohorts provide evidence of structural and functional alterations in the CNS. A structural MRI study conducted by Rothstein has identified a depletion in cortical grey matter volume in long COVID patients experiencing neurological symptoms [187]. Similarly, Diez-Cirarda et al. findings revealed persistent structural and functional abnormalities in the brains of people with long COVID after 11 months of SARS-CoV-2 infection [188]. Interestingly, the authors reported hypoconnectivity between left and right parahippocampal areas, and between bilateral orbitofrontal and cerebellar areas along with loss in grey matter volume in cortical, limbic and cerebellar areas [188]. Furthermore, the reduction of grey matter volume was significantly associated with neurocognitive dysfunction [188]. A recent study also found structural and microstructural changes in multiple cortical and subcortical regions, including cortical thinning in the cingulate and insular cortices, increased susceptibility in the hippocampus, and enlarged choroid plexus volume in long COVID individuals with cognitive impairment [189].

A recent investigation by Serrano Del Pueblo et al. identified brain structural changes associated with reduced overall cognitive levels in people with long COVID compared to a group of convalescent COVID-19 controls [190]. The investigators found that 48% of patients with long COVID reported episodic memory deficit, with 27% also reporting impaired overall cognitive function (mainly attention, working memory, processing speed and verbal fluency) [190]. Most importantly, structural MRI analysis identified structural alterations that may underlie these neurocognitive deficits in long COVID. These alterations include cortical thinning in the left posterior superior temporal gyrus and widespread white matter alterations, including lower fractional anisotropy and higher radial diffusivity compared with controls [190]. Significantly larger volumes in left hippocampal subfields, including the subiculum head, presubiculum head, molecular layer, and whole hippocampal head have been reported in long COVID patients compared with healthy controls [30]. These findings were associated with the severity of cognitive (fatigue, impaired concentration and unrefreshing sleep) and physical symptoms (pain and physical function) and illness duration, suggesting that hippocampal remodelling may underlie persistent neurocognitive dysfunction in long COVID, which may reflect compensatory or adaptive responses to chronic symptom burden [30]. Memory performance, integration and delayed recall depend on specialised subfields within the hippocampus. These subfields decline in the neurodegenerative conditions [191–193]. These structural alterations may reflect increased neurogenesis or functional compensation and could contribute to the persistent cognitive dysfunction observed in long COVID [30].

Functional MRI (fMRI) provides an opportunity to link persistent brain changes and functional connectivity with symptoms of long COVID. Research conducted by Troll et al. demonstrated significantly increased functional connectivity between the right caudate nucleus and both the left and right precentral gyri in people with long COVID compared with age- and sex-matched controls, and this connectivity also correlated with cognitive assessment scores [178]. This finding is similarly supported by Leitner et al., who reported elevated functional connectivity between the default mode network and parts of the posterior supramarginal gyrus, angular gyrus and posterior-occipital middle temporal gyrus associated with cognitive impairment [194]. Barnden et al. reported altered connectivity in between the midbrain and left rostral medulla in long COVID compared with healthy controls using 7 Tesla MRI [179]. Additionally, Dacosta-Aguayo et al. detected increased functional connectivity among the bilateral hippocampus, the right hippocampus and the left amygdala, and the right hippocampus and the left middle temporal gyrus in the long COVID group [195]. Research using fMRI has reported on substantial changes in functional connectivity in various brain regions. Studies have described a significant correlation between connectivity changes in neurocognitive symptoms of long COVID, emphasising the clinical implications of neurodegeneration. While neurodegenerative diseases are referred to as disconnection syndromes with an overall reduction in functional connectivity across brain networks, increases in connectivity are observed as compensatory mechanisms, as reported in Alzheimer’s disease [196, 197].

An MRI study with diffusion tensor imaging (DTI) observed a negative correlation between memory performance and increased radial diffusivity throughout various white matter tracts, similarly in both hemispheres in people with long COVID [195]. This demonstrates evidence of memory impairment associated with lower white matter network integrity, and higher radial diffusivity in long COVID, suggesting neuroinflammation or diminished myelination of axons. Moreover, it is another overlap with multiple sclerosis neurological findings, given that radial diffusivity is frequently increased in patients with multiple sclerosis, which is associated with damage to nerve cells and cognitive impairment [198, 199]. White matter changes were also observed in a study conducted by Diez-Cirarda et al., which also showed reduced mean diffusivity and axial diffusivity in the corpus callosum, forceps minor, middle longitudinal fasciculus, uncinate tract and fronto-occipital fasciculus in the long COVID cohort [188]. However, no significant differences were observed regarding fractional anisotropy and radial diffusivity between long COVID and control groups in specific brain tracts [188]. Fatigue was significantly correlated with diminished CBF in the right occipital regions in a cohort of long COVID but did not find a correlation with cognitive and olfactory performance [200]. Additionally, reduced CBF in the left frontal-temporal gyrus was associated with an increase in fatigue and a decrease in cognition in people with long COVID in another study [201]. Despite the conflicting findings regarding blood perfusion in the brains of individuals with long COVID, the consistent evidence of neuroinflammation, microvascular thrombosis, and BBB disruption in long COVID pathogenesis is proposed to account for disturbances in CBF and contribute to neurological symptoms.

An experiment conducted using high-resolution magnetic resonance spectroscopy (MRS) detected reduced total choline levels in the dorsal anterior cingulate cortex in people with long COVID, which is a notable observation that may relate to blood clotting mechanisms being linked to brain fog symptoms [27]. It is noteworthy in the context of blood clotting in long COVID patients to mention that an animal study has found a neuroprotective effect of choline administration to prevent disseminated intravascular coagulation [202]; therefore, the choline deficiency is perhaps associated with coagulation disturbances in long COVID. Furthermore, lower plasma choline was associated with an increased risk of cognitive impairment in post-stroke patients [203]. Additionally, another study investigating neurochemical changes in long COVID, recently reported that glutamate and N-acetyl-aspartate levels were significantly elevated in the posterior cingulate cortex compared with healthy controls, which is linked to symptom severity, suggesting a link between biochemical changes and cognitive or fatigue-related symptoms [29]. Disturbances to brain metabolites may result in microenvironment changes resulting in increased oxidative stress in neuronal damage in people with long COVID.

In addition to neuroimaging techniques, blood proteomics may uncover evidence of neurodegeneration. For instance, an investigation by Seo et al. investigated blood proteins and brain MRI from individuals with long COVID 12 months following acute mild COVID-19 [189]. Specific blood proteins assessed included glial fibrillary acidic protein (GRAP), neurofilament light (NFL), p-Tau181, and the Aβ40/42 ratio. Significantly higher GFAP levels were observed in people with long COVID experiencing cognitive disturbances compared with other long COVID symptomatic cohorts. Further, GFAP and NFL levels were correlated with evidence of cognitive dysfunction. In conjunction with brain MRI findings such as enlarged choroid plexus among other aberrations, the study suggested the results are indicative of increased neuronal and astroglial damage, which may be associated with neurodegeneration processes.

The convergence of brain MRI findings and blood proteomics provides insight into the disease pathomechanism, as well as an opportunity for biomarker discovery and the development of screening tools to assess risk and clinical outcomes of long COVID. Importantly, long COVID is an emerging condition with a limited research history. Consequently, longitudinal studies are essential to validate existing findings, uncover underlying mechanisms, and track disease progression across patients with varying severity levels. Supplementary Table 1 summarises the methodology, main findings and limitations of the studies discussed in this section.

## Clinical implications

Neurodegenerative diseases are hallmarked by progressive neuronal loss, commonly manifesting in older age, and represent a significant global burden given the profound impact on disability and mortality rates [204, 205]. Parkinson’s disease, Alzheimer’s disease, dementia, multiple sclerosis and Huntington’s disease are among the most prevalent and extensively studied neurodegenerative disorders [122, 206, 207]. Long COVID has received increasing attention, given the overlap in clinical features (such as cognitive issues, sleep disturbance, mood changes, autonomic dysfunction, pain) with neurodegenerative diseases, as well as reports of exacerbated clinical manifestations in patients with pre-existing Parkinson’s and Alzheimer’s diseases following SARS-CoV-2 infection [32, 208–210].

There are numerous pathways through which SARS-CoV-2 may result in neurological symptoms, as well as structural and functional changes in the brain in individuals diagnosed with long COVID [211]. In addition, multiple biological mechanisms may be implicated in the development of neurodegenerative sequelae in long COVID, alongside shared underlying risk factors in individuals infected with SARS-CoV-2 [156]. To date, some evidence indicates SARS-CoV-2 may damage neurons and contribute to the initiation of degenerative processes underlying long COVID symptoms or exacerbate pre-existing neurodegenerative diseases [11]. However, current data remain preliminary and lack longitudinal evidence. Consequently, we cannot yet definitively determine whether prior SARS-CoV-2 infection triggers an irreversible pathway that accelerates neurodegeneration in the CNS. Interestingly, findings from a large US Department of Veterans Affairs cohort published by Xu et al. identified increased risk of diverse neurological complications emerging after 12 months from acute COVID-19, including stroke, cognition and memory disorders, peripheral nervous system disorders, migraines, seizures, mental health disorders, musculoskeletal disorders, sensory disorders, Guillain–Barré syndrome, conditions affecting motor coordination and movement encephalopathy [4]. SARS-CoV-2 infection is suggested to have the potential to increase the likelihood of developing Alzheimer’s disease, Parkinson’s disease or multiple sclerosis six months following infection, compared with other respiratory infections [212]. Furthermore, people with pre-existing neurodegenerative diseases are of higher susceptibility to contract SARS-CoV-2 and have an increased risk of developing long COVID given pre-existing neuroinflammatory responses. A study reported that people with Alzheimer’s disease or Parkinson’s disease were 40% more likely to experience sequelae after COVID-19 [4]. Although there is growing research suggesting the contribution of SARS-CoV-2 in the development of neurodegenerative conditions associated with long COVID, further investigation is required to validate and analyse the long term the impact of the virus on people recovered or not from the acute SARS-CoV-2 infections.

Based on the findings from neurodegenerative diseases research and the emerging evidence from long COVID, neurodegeneration may present substantial clinical implications in the presentation of symptoms already discussed, as well as structural brain changes and altered functional connectivity [29, 178, 188, 213]. Longitudinal investigations of neurodegeneration in long COVID are essential for effective disease monitoring, biomarker development and identification of therapeutic targets. Furthermore, neurocognitive and brain imaging alterations correlate with clinical presentation in people with long COVID and reduced quality of life, emphasising healthcare burden. Poor clinical outcomes may further predispose individuals with long COVID to neurodegeneration and heightened risk of developing secondary diseases.

Given SARS-CoV-2 is a new virus that emerged recently, the long-term effects on human health remain incompletely understood. It is also critical to note that aging is the most consistent risk factor associated with neurodegenerative processes. Hence, longitudinal studies, especially those following participants for decades, are crucial for elucidating any potential contribution of SARS-CoV-2 with neurodegeneration. Moreover, it is essential to distinguish effects in individuals diagnosed with long COVID and those people completely recovered from the acute infection. Additionally, there is a lack of biomarkers, which presents ongoing challenges, including limitations in achieving an adequate diagnosis and disease prognosis, difficulties in identifying the pathomechanism of long COVID, and reduced reproducibility of research findings.

## Potential limitations and confounding factors in neurological research in long COVID

The heterogeneous nature of long COVID patients over studies, characterised by a wide variety of clinical symptoms, consequently leads to diverse clinical subgroups within the affected population. A confounding factor in long COVID research, and therefore also influencing the findings described in this review, is the categorisation of long COVID patients using different approaches based on symptom occurrence, affected organ systems, long COVID severity and clinical indicators [214]. Although the term long COVID has been widely used in the literature, there is no universally accepted definition, which has a significant impact on research [215]. In the context of long COVID, case definitions are conceptually similar but vary in aspects such as illness duration and symptom clustering, leading to distinct participant subgroups [38]. For instance, WHO Delphi consensus indicates post COVID-19 condition occurs three months from the onset of SARS-CoV-2 infection, with symptoms for at least two months and not explained by another diagnosis [216]. While the National Institute for Health and Care Excellence (NICE) defines post-COVID-19 syndrome as being continuous for more than 12 weeks, but defines the term ‘long COVID’ as ongoing symptomatic COVID-19 (4–12 weeks) and post-COVID-19 syndrome (≥ 12 weeks) [217]. Other recognised institutions, such as Centers for Disease Control and Prevention and the National Academies of Sciences, Engineering, and Medicine, also have their own specifications for case definitions; therefore, research on long COVID has adopted different criteria, which may explain divergence in results, as participant characteristics vary depending on the criteria applied.

Additionally, the heterogeneity of long COVID extends to the study populations in this review, which vary by potential confounding factors such as age, sex, hospitalisation status, infection duration and severity, vaccination status, SARS-CoV-2 variants, acute treatment medications, and comorbidities. Hospitalisation status is a factor that should be considered in the data analysis of studies in the context of long COVID, as a high number of patients require hospitalisation, including intensive care unit admission during acute COVID-19. Similarly, vaccination status directly affects the investigation findings, as it has been well established that vaccines play a protective role in minimising the impact of long COVID [218, 219], however, most studies did not report information regarding this data, which is a limitation previously discussed in other reviews [220, 221]. Studies evaluating the prevalence or correlating demographic features with severity or prognosis may achieve different results, depending on whether they adjust the data for factors such as those discussed in this section. Further limitations of the evidence are associated with the methodological process, such as study design, follow-up duration and measurement methods. The neuroimaging studies included in this review, as well as those in the current literature, use a cross-sectional design including structural and functional alterations (e.g. articles included in the Supplementary Table 1). Findings from cross-sectional designs might be affected by inter-individual variability due to the clinical heterogeneity and other confounding factors as discussed above.

Overall, emerging findings on neurological disruptions in long COVID, including those related to the neurodegenerative process, are characterised by substantial heterogeneity, which requires caution to interpret results. This variability, alongside other limitations and potential confounding factors, may explain the conflicting findings identified in this review and the divergent outcomes reported across studies.

## Conclusion

The potential neurodegeneration following SARS-CoV-2 infection may be multifactorial and long COVID is a complex condition with mechanisms that remain elusive. Evidence to date indicates SARS-CoV-2 persistence, neuroinfiltration of the virus, neuroinflammation, dysregulated BBB permeability and microvascular injury may play a role in neurodegenerative processes in long COVID. Research has documented neurodegeneration in long COVID, such as significant microglial activation, structural and functional alterations. However, in relation to long COVID, conclusions must be interpreted cautiously, given that the evidence is limited to short-term outcomes.

Understanding the mechanisms underlying neurodegeneration in long COVID is essential for mitigating long-term neurological consequences and for developing screening strategies, including identifying risk factors and biomarkers. Finally, characterising the neurodegenerative processes associated with long COVID is crucial for clinical practice, as it enhances differential diagnosis from other neurodegenerative disorders and improves treatment strategies. In the future, longitudinal investigations may establish causality and progression of long COVID and provide more consistent data to analyse the neurodegeneration associated with long COVID. Research into the role of virally induced neurodegeneration in neurodegenerative diseases including multiple sclerosis and Alzheimer’s disease provides an opportunity to learn from previous knowledge. Recognising the complexity of long COVID and the current limitations, however, this review discusses preliminary findings and highlights the critical need for continued research to enhance understanding and potentially mitigate long-term impacts in the following decades.

## Supplementary Information

Below is the link to the electronic supplementary material.

Supplementary Material 1

## Data Availability

Not applicable.
