# Supplementary material for: Neurological impairment in long COVID: implications for neurodegenerative disease
Source: J Transl Med. 2026 Jul 10;24:1248. doi: 10.1186/s12967-026-08607-y (PMC13640182; doi:10.1186/s12967-026-08607-y)
Supplement: Supplementary file 1 [file 12967_2026_8607_MOESM1_ESM.docx]

**Supplementary Table 1. Neuroimaging studies in long COVID**

| **Study** | **Imaging Modality** | **Location and Study period** | **Cohort (n cases / n controls)** | **Timing post-infection** | **Long COVID Definition** | **Major Findings** | **Key Limitations / Confounders** |
| --- | --- | --- | --- | --- | --- | --- | --- |
| **Guedj et al. (2021)** | ¹⁸F-FDG brain PET | French,  May 2020 -  September 2020 | 35 long COVID patients; 44 age/sex-matched HC | Mean 97 days (range 26–155 days) post symptom onset | History of confirmed SARS-CoV-2 infection with persistent  fatigue, ≥3 weeks after confirmed SARS-CoV-2 infection (PCR or serology); excluded patients with encephalitis/stroke, neurologic/psychiatric antecedents. | Bilateral hypometabolism in: rectal/orbital gyrus (including olfactory gyrus), right temporal lobe (amygdala, hippocampus, extending to the right thalamus), bilateral pons/medulla and cerebellum. Hypometabolism is associated with more functional complaints (brainstem and cerebellar clusters) and with symptom (hyposmia/anosmia, memory/cognitive impairment, pain and insomnia). | Retrospective, monocentric; most qualitative symptom assessment (no validated scales); 39% ICU-hospitalised patients and 16% a mechanical ventilation; small subgroups of patients report use of ACE and/or nasal spray analyses; no baseline pre-infection. |
| **Verger et al. (2022)** | ¹⁸F-FDG PET (visual interpretation classifying as normal, mildly, moderately or incompletely affected, or severely affected within hypometabolic pattern) | France, August 2021 - October 2021 | 143 patients referred for suspected neurological long COVID (68.53% female; mean age 47.4±13.6 years) | Mean 10.9± 4.8 months from symptom onset | WHO case definition (Delphi consensus); previous positive RT-PCR and/or serology test for SARS-CoV-2 | Visual interpretation: 53% of scans normal, 21% mildly, moderately or incompletely affected, 26% severely affected. Typical hypometabolic pattern: fronto-orbital/olfactory regions, brainstem, cerebellum, other limbic and paralimbic regions (including amygdalae, hippocampal and parahippocampal regions). | No quantitative analysis (visual only); no HC group; unequal participant across different study centres (Nancy=10, Paris=29 and Marseille=104 individuals) variability; no pre-infection baseline; no investigation of relationships between symptoms and brain PET findings. |
| **Barnden et al. (2023)** | Task fMRI (7T; Stroop colour-word task) | Australia, the period was not specified | 10 long COVID (7 women, 3 men; mean age 44±15 years); 13 HC (7 women, 6 men; mean age 39±13 years) | Not mentioned | WHO working case definition (Delphi consensus): ≥1 moderate-to-severe symptom onset ≤3 months after COVID-19 infection, persisting ≥3 months | Increased functional connectivity (long COVID > HC) between the medulla and midbrain. Most differences were in the brainstem and salience network. Imparied functional connectivity correlated with disability, heart rate variability, and respiration variability. | Limited number of subjects (n=10 long COVID, n=13 HC); large sample study required to confirm results. |
| **Díez-Cirarda et al. (2023)** | Multimodal functional and structural MRI (3T): resting-state fMRI, VBM (T1), DTI, WM lesion segmentation | Spain, November 2020 - December 2021 | 86 post-COVID syndrome patients with subjective cognitive complaints (67.44% female; mean age 50.71±11.20 years; 29 hospitalised, 8 ICU); 36 HC (61.11% female; mean age 49.33±15.99 years) | Mean 11.08±4.47 months post first SARS-CoV-2 symptoms | PCR-confirmed COVID-19 ≥3 months before inclusion; subjective cognitive complaints temporally related to infection; no prior neurological/psychiatric disorders | Brain functional and structural abnormalities. Reduced FC between bilateral parahippocampal gyri, and between left cerebellar vermis and bilateral frontal superior orbital cortex. WM: reduced axial and mean diffusivity (right-lateralised) in corpus callosum, forceps minor, middle longitudinal fasciculus, uncinate tract and fronto-occipital fasciculus. GM atrophy correlated with attention, working memory and processing speed deficits. | Cross-sectional; cognitive/mood not assessed in controls. |
| **Rothstein (2023)** | Structural MRI (3T): 3D Voxel-Based Morphometry (NeuroQuant® automated software) | United State of America, the period was not included | 24 consecutive COVID-19 infected patients with long term neurologic symptoms (79% females, mean age 46.9 years; all mild respiratory illness, no hospitalisation or ventilation required); compared to NeuroQuant® normative database (age/sex-matched controls) | Median 85 days after laboratory confirmation of COVID-19 infection | WHO case definition (Delphi consensus) and NICE guideline: neurological symptoms lasting ≥8 weeks, PCR-confirmed SARS-CoV-2; no prior neurological impairment or chronic fatigue | Depletion of cortical grey matter volume. Thalamus volume significantly elevated. No significant difference in whole brain, hippocampal or forebrain parenchyma volume vs normative data. Most common symptoms: cognitive impairment/brain fog (91.6%), fatigue (87.5%), new headache (41.6%), word-finding difficulty (41.6%). | Small sample size; comparison to normative database (not prospective controls); mild acute illness only (outpatient); predominantly female sample (79%); self-reported neurological complaints only; patients evaluated at cognitive disorders clinic (selection bias toward cognitive impairment); no vaccination or treatment data; likely heterogeneous COVID-19 variants, and did not provide period of investigation; single-centre. |
| **Dacosta-Aguayo et al. (2024)** | Multimodal MRI (3T): DTI and resting-state fMRI, resting-state functional connectivity (rs-FC) and correlations with TBSS and resting state | Spain, January 2022 - March 2023 | 53 long COVID patients with cognitive complaints (88.7% female; mean age 48.23±9.2 years; 81.1% mild-moderate COVID-19; 9 hospitalised, none ICU) | Mean 1.8±0.52 years after COVID-19 infection diagnosis | WHO case definition (Delphi consensus), persistent cognitive complaints after SARS-CoV-2; excluded prior psychiatric/neurological conditions that could affect cognitive functions, history of substance abuse or life expectancy ≤ 6 months. | DTI: increased radial diffusivity (RD) across bilateral white matter tracts negatively correlated with memory performance.  rs-fMRI: greater activation in right fronto-parietal network (right MTG, bilateral PCC) inversely correlated with memory. Hippocampal functional connectivity increased in bilateral hippocampus, right hippocampus and the left amygdala, and the right hippocampus and the left MTG, FC were inversely correlated with memory but was not significant after adjusting for multiple comparison correction | No HC group (group correlations only); small sample size; predominantly female sample (88.7%); cognitive domains and MRI data collected in sessions within 6 months apart. |
| **Serrano del Pueblo et al. (2024)** | Structural MRI (1.5T): cortical thickness (FreeSurfer); DTI/TBSS (FSL) | Spain, the period was not included | 83 long COVID patients with neurological symptoms (mean age 50.82 ± 1.03 years, 71% female); 22 infection-recovered controls (mean age 50.50 ± 2.59 years, 36% female), matched for age, sex, education | Long COVID = 16±1 and controls = 15±2 months from the acute infection | No long COVID case definition specified. Neurological long COVID: clinical diagnosis of neurological long COVID (including evidence of past infection with COVID-19); persistent neurological symptoms >2 months after COVID-19, assessed by neurology department; PCR/antigen-confirmed past infection | Cortical thinning in left posterior superior temporal gyrus and widespread white matter abnormalities (reduced fractional anisotropy, increased radial diffusivity) indicative of poorer white matter integrity in long COVID vs. controls. Fractional anisotropy reductions correlated with lower performance across multiple cognitive domains; 48% of patients showed episodic memory deficits, with over a quarter also impaired in attention, working memory, processing speed, and verbal fluency. | No long COVID case definition was specified; 1.5T scanner (lower resolution than 3T/7T); higher proportion of females in patient group (71% vs 36% in control group). |
| **Troll et al. (2025)** | Resting-state fMRI (3T; seed-based FC analysis) | Germany, the period was not included | 60 long COVID patients (mean age was  45.38±13.33 years, 37 women, 7 hospitalised and 3 received oxygen therapy); 52 age- and sex-matched HC (mean age 41.19±11.17 years, 30 women, 50% prior COVID-19 infection confirmed by PCR) | Varies from 8 weeks to 16 months post-acute  COVID-19 | Not specified. Inclusion criteria include one of three neuropsychiatric complaints  (fatigue, lack of concentration, and depressed mood), an  intelligence quotient higher than 80, and duration of symptomatology for at least four weeks beyond the acute infection of COVID-19 | Increased functional connectivity between right caudate nucleus and bilateral precentral gyri. Increased FC between right anterior globus pallidus and posterior cingulate cortex and right temporal pole. The magnitude of FC between the caudate and the left precentral gyrus were negatively correlated with MoCA score and Trail Making Test B. Cognitive impairment severity correlated with age, sex, and severity of depression but not with initial infection severity or symptom duration. | Broad heterogeneity of disease duration, long COVID definition not specified;  limited hospitalised patient representation;  limited sensitivity of MoCA to detecting cognitive impairment in younger adults. |
| **Godlewska et al. (2025)** | ¹H MRS (7T; brain: pgACC and dACC; calf muscle) | United Kingdom, February 2020 - July 2022 | 25 long COVID (mean age 44.3 years, 19 females); 24 HC (mean age 40.5 years, 12 females; 24 ME/CFS (mean age 32.6 years, 16 females) | Long COVID: Mean 15.3 months since onset | Clinician-diagnosed long COVID (≥ 2 symptoms: high fever, shortness of  breath, persistent cough, loss of taste or smell); new fatigue post COVID-19 persisting ≥12 weeks, present at assessment and impairing daily functioning. Exclusion of current or previous psychiatric conditions and  comorbidities that affect energy processing. | Long COVID: reduced total choline in dACC than HC; ME/CFS: elevated lactate in both pgACC and dACC than HC. | Small sample sizes; no multiple comparison correction (exploratory); only inclusion of mild-to-moderate patients (ambulatory); antidepressant use in 24% of long COVID and 20.83% ME/CFS patients; age differed significantly between groups; Missing data due to technical and data quality issues |
| **Hosp et al. (2025)** | 3T-MRI: Arterial Spin Labelling (ASL) for cerebral blood flow (CBF); FreeSurfer cortical morphometry | Germany, June 2020 - October 2022 | 55 Post-COVID Condition (PCC) patients (median age 39 years; 68% female); 36 COVID-recovered controls without PCC (median age 44 years; 67% female) | PCC median 319 days after positive PCR test for SARS-CoV-2; controls median 227 days post-PCR | WHO case definition (Delphi consensus); PCR-confirmed SARS-CoV-2 infection previously; neurocognitive symptoms. Exclusion of pre-existing neurodegenerative disorders | No significant whole-brain or regional CBF differences after FDR correction. At exploratory threshold: trend for CBF reduction in right angular gyrus and right middle occipital gyrus in PCC vs controls. Significant inverse correlation between fatigue and CBF in right middle and inferior occipital gyri. The effect was significantly associated with physical fatigue, not with mental fatigue. | Explorative CBF findings did not survive FDR correction; fatigue measured by WEIMuS (originally validated for Multiple Sclerosis). |
| **Leitner et al. (2025)** | Structural and resting-state fMRI (3T) | Austria, October 2022 - February 2023 | 39 long COVID patients (84.6% females, mean age 49.97±8.63 years) split into: cognitively impaired (n=16) and cognitively unimpaired (n=23) | Mean 18.08±10.42 months since disease onset | WHO case definition (Delphi consensus), that caused new health impairment or deterioration of a pre-existing disease. Excluded dementia, Multiple Sclerosis, Parkinson's, stroke | Within-network FC: cognitively impaired showed significantly higher FC between DMN and posterior supramarginal gyrus, angular gyrus and right temporo-occipital and middle temporal gyrus. Between-network FC: cognitively impaired had significantly stronger connectivity between occipital pole (visual network) and ventral stream network. No significant differences in the structural alteration between groups. | No HC group; small sample per group; broad cognitive grouping (not domain-specific); predominantly female sample (84.6% female). |
| **Martini et al. (2025)** | ¹⁸F-FDG PET scan | Italy, October 2020 - May 2023 | 44 neuro-COVID patients (mean age 69.31±12.25 years; 50% female) classified as: acute (n=7, <4 weeks), subacute (n=17, 1–3 months), long-term (n=20, 4–12 months) post-infection; 125 HC (AIMN database; mean age 65.78±11.33 years; 53.6%) | Acute: <4 weeks post-infection; subacute: 1–3 months; long-term/chronic: 4–12 months post-infection | WHO case definition (Delphi consensus); Confirmed SARS-CoV-2 infection by RT-PCR; new-onset cognitive impairment or neurological signs in context of acute/subacute or long COVID not explained by alternative diagnosis; no prior neurological/psychiatric history; FDG-PET performed within 2 weeks of clinical assessment | Fronto-insular hypometabolism (orbitofrontal cortex, superior/inferior/middle frontal gyri, medial prefrontal cortex, insula, angular gyrus) was identified across all 44 patients. Hypometabolism severity and extension decreased significantly from acute to subacute to long-term phases; Hypometabolism decreased from acute to long-term phases. Network connectivity (IRCA): acute/subacute phase — hyperconnectivity in Executive Network (EXN +27%) and Attention Network (ATTN +20%), Anterior Salience Network (ASN, +25%), Limbic Network (LIN, +26%). Long-term phase — hypoconnectivity in EXN (–41%) and ATTN (–27%); ASN and LIN normalised toward HC. | Only one patient evaluated longitudinally (acute + 5-month follow-up); MMSE used for cognitive screening (not sensitive for subtle deficits); HC from external database (not matched on scanner or acquisition protocol). |
| **Seo et al. (2025)** | Multimodal MRI (3T): cortical thickness (FreeSurfer), paramagnetic susceptibility, choroid plexus volume, DTI-ALPS index; blood proteomics (Simoa: GFAP, NfL, Aβ42/40, p-Tau181; Olink Explore 3072 panel) | South Korea, November 2022 - October 2023 | Exploration cohort: 160 participants (Cog-PASC n=28, Other-PASC n=55, NS-PASC n=77); covariate-matched sub-cohort n=82 (Cog-PASC n=28, Other-PASC n=27, NS-PASC n=27); independent validation cohort: 109 participants (Cog-PASC n=16, Other-PASC n=47, NS-PASC n=46). Predominantly non-hospitalised, Omicron-dominant infection. | Exploration cohort: median median of 13.0 months [IQR 8.0–15.0] post-infection.; validation cohort: median 18.0 months [IQR 11.0–23.0] post-infection | No long COVID case definition specified. Cog-PASC: MoCA <26 (mild cognitive impairment). Other-PASC: MoCA ≥26 with FAS ≥22 (elevated fatigue) and/or PASC symptom score ≥12. NS-PASC: no significant PASC symptoms. SARS-CoV-2 antibody confirmed. | . Cortical thickness: Cog-PASC showed thinning in cingulate cortex (anterior and posterior), insular cortex, and parahippocampal gyrus vs Other-PASC; replicated in validation cohort with additional frontal/parietal involvement. Paramagnetic susceptibility (iron deposition): increased in cingulate, occipital cortex, and hippocampus in Cog-PASC; confirmed in validation cohort. Choroid plexus volume fraction: significantly larger in Cog-PASC vs Other-PASC and NS-PASC; DTI-ALPS index: significantly reduced in Cog-PASC in validation cohort (suggesting impaired glymphatic function). WM TBSS: elevated MD/RD and reduced fractional anisotropy across bilateral tracts in Cog-PASC (validation cohort). | Cross-sectional preliminary report from ongoing longitudinal study; most participants included were non-hospitalised (~98%); exploration and validation cohorts not fully comparable (enrolled at different times, different follow-up durations); age/sex/education imbalance between groups (adjusted for, but residual confounding possible). |
| **Thapaliya et al. (2025)** | 7T MRI: structural hippocampal subfield volumetry (FreeSurfer) | Australia, July 2021 – August 2023 | 17 long COVID (mean age 51.65±11.26, 73.3% females); 29 ME/CFS (mean age 43.31±11.24 years, 79.3% females); 15 age-matched HC (mean age 38.26±12.74, 66.7% females) | Disease duration long COVID (0.60±0.46 years) | WHO case definition (Delphi consensus); (ME/CFS patients met Canadian Consensus Criteria and/or International Consensus Criteria and had no COVID-19 history) | Both long COVID and ME/CFS showed larger left hippocampal subfield volumes compared to HC (subiculum head, presubiculum head, molecular layer, whole hippocampal head). In long COVID, hippocampal volumes correlated negatively with fatigue, unrefreshing sleep, pain, and illness duration. | Small sample size (limits sex/handedness subgroup analyses); clinical severity measures obtained via online self-report surveys (potential for under/over-reporting). |
| **Thapaliya et al. (2025b)** | Multimodal MRI (3T): T1w/T2w ratio mapping (myelin signal); DTI/TBSS; Magnetic Resonance Spectroscopy (MRS) in posterior cingulate cortex | Australia, the period was not included | 19 long COVID (mean age 47.95±13.28 years, 73.7% female); 12 COVID-recovered HC (COVID-RHC, mean age 34.76±7.10 years, 75% female); 16 HC without SARS-CoV-2 infection (Non-COVID-HC, mean age 38.85±12.53 years, 68.8% female) | Mean illness duration 0.78±0.69 years for long COVID | WHO case definition (Delphi consensus) | T1w/T2w signal was elevated in long COVID vs. Non-COVID-HC in precentral and middle temporal gyrus, and in COVID-recovered vs. long COVID across multiple white matter tracts, brainstem, and cerebellum. DTI showed lower mean diffusivity in dentate regions in long COVID vs. Non-COVID-HC, and higher fractional anisotropy in right SLF in long COVID vs. COVID-RHC; COVID-RHC showed lower diffusivity metrics in left caudate and right supramarginal gyrus vs. Non-COVID-HC. MRS revealed higher N-Acetylaspartate in long COVID and higher glutamine in COVID-RHC in posterior cingulate cortex. T1w/T2w signal in middle temporal gyrus correlated positively with physical function, and in midbrain negatively with cognitive dysfunction. | Small sample size; significant age difference between long COVID and COVID-RHC groups; exploratory statistical thresholding (uncorrected voxel-wise p<0.001) may introduce Type I error. |
| **Wang et al. (2025)** | 3T MRI: Arterial Spin Labelling (ASL) for cerebral blood flow (CBF); imaging transcriptomics (Allen Human Brain Atlas) | China, HC and COVID-19 recovered = October 2021 - June 2022, long COVID = January - April 2023 | 22 long COVID (mean age 54.18±8.57 years, 54.5% female); 50 COVID-19 recovered (mean age 51.3±8.20 years, 54% female); 30 HC (pre-outbreak, mean age 54.30±2.37 years, 70% female) | Median long COVID 90 days (IQR: 58–110 days) and COVID-19 recovered 110.00 days (IQR:76.75–116.50) post-infection | NICE guideline: persistent, recurring, or new symptoms >4 weeks after acute COVID-19 infection, not attributable to other illnesses. Excluded those who had a history of CNS diseases, psychiatric disorders, or first-degree relatives with psychiatric disorders | Long COVID showed reduced CBF in left inferior frontal/superior temporal/middle frontal gyrus vs. HC; COVID-19 recovered group showed similar reductions in bilateral medial/superior frontal gyrus and supplementary motor area. Lower CBF correlated with greater fatigue and poorer immediate memory in long COVID. 2,178 genes spatially linked to CBF changes, enriched in mitochondrial/energy pathways, COVID-19, and neurodegeneration; 51 overlapped with 332 SARS-CoV-2 protein-interacting genes, with expression concentrated in oligodendrocyte and astrocyte lineages. | Small sample; transcriptomic and neuroimaging data from different cohorts; left hemisphere only analysed for gene expression; symptom heterogeneity at time of scanning may affect gene expression. |

*Abbreviations: ACC, anterior cingulate cortex; AIMN, Associazione Italiana di Medicina Nucleare ed Imaging Molecolare; ASL, arterial spin labeling; ATR, anterior thalamic radiation; CBF, cerebral blood flow; Cog-PASC, group with cognitive impairment; dACC, dorsal ACC; DAN, dorsal attention network; DMN, default mode network; DTI, diffusion tensor imaging; FC, functional connectivity; FDG, fluorodeoxyglucose; fMRI, functional magnetic resonance imaging; GM/GMV/GMC, grey matter/volume/concentration; HC, healthy controls; ICU, intensive care unit; IFOF, inferior fronto-occipital fasciculus; ILF, inferior longitudinal fasciculus; IRCA, interregional correlation analysis; MoCA, Montreal Cognitive Assessment; MRS, magnetic resonance spectroscopy; MTG, middle temporal gyrus; NfL, neurofilament light chain; NICE, National Institute for Health and Care Excellence; NS-PASC, no significant PASC group; Other-PASC, group with multifaceted long-COVID symptoms other than cognitive impairment; PASC, post-acute sequelae of SARS-CoV-2; PCC, posterior cingulate cortex / Post-COVID Condition; PCR, polymerase chain reaction; pgACC, pregenual ACC; RD, radial diffusivity; rs-fMRI, resting-state fMRI; SLF, superior longitudinal fasciculus; TBSS, tract-based spatial statistics; 3T, 3 Tesla; 7T, 7 Tesla; VBM, voxel-based morphometry; WEIMuS, Würzburg Fatigue Inventory in Multiple Sclerosis; WM, white matter; WMH, white matter hyperintensities.*
